# Evaluation of the Amblyopia tracker app

**DOI:** 10.1007/s00417-024-06413-0

**Published:** 2024-02-27

**Authors:** Anna R. O’Connor, Ashli Warburton, Martha Farrelly-Waters, Laura England, Rachel Clarke, Hazel Kay

**Affiliations:** 1 School of Health Sciences, University of Liverpool, Thompson Yates Building, Brownlow Hill, Liverpool, L69 3GB UK; 2 Orthoptic Department, Manchester Royal Eye Hospital, Manchester, UK; 3 Kay Pictures, Tring, UK

**Keywords:** Amblyopia, Visual acuity, App, Vision, Telehealth

## Abstract

**Purpose:**

The Amblyopia tracker app has been developed to be a tool for parents to monitor changes in vision at home during amblyopia treatment. The aims of this study were to evaluate the feasibility and repeatability of parents testing their children at home and to compare home test results to an assessment in clinic by an orthoptist.

**Methods:**

Children (age < 18 years) with amblyopia (interocular acuity difference of ≥ 0.2logMAR) were recruited. Parents were asked to test their child with the app three times during a two week period followed by an online questionnaire about the usability. Participants also tested within 48 h of their appointment where the measurement was repeated by an orthoptist.

**Results:**

Out of 277 potential participants contacted, 37 completed three home measurements, mean age 6.8 years (SD 2.94). Home tests comparisons were made between test two and three to ensure familiarity with the process. Paired *t*-tests showed no statistically significant difference for either eye or the interocular acuity difference (IAD). However, 29% had a difference in IAD of more than 0.1logMAR on repeated testing, with a maximum of 0.4logMAR difference in the IAD. Questionnaire responses from the parents who participated were predominantly positive with 97% of respondents saying they would use it if were available. Comparison of home and clinical measurements (*n* = 23, mean age 6.72 SD 2.60) showed no statistically significant differences for either eye or interocular acuity difference (paired* t*-test, *p* > 0.3 in all cases).

**Conclusion:**

Results show no statistically significant differences for the Amblyopia tracker app when used by parents at home on repeated testing, or between the home test by a parent and the test by a clinician. However, variability in the results does indicate that further improvements are required to ensure the results can be used as a reliable clinical tool.

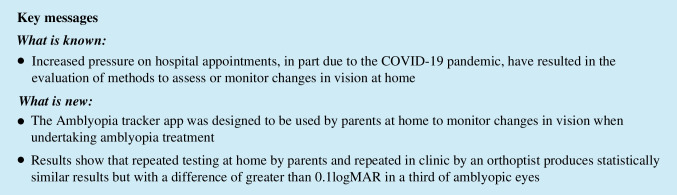

## Introduction

Telehealth has been a growing aspect of all health care for many years but was accelerated during the COVID-19 pandemic. However, in paediatric ophthalmology, the majority of clinic care still involves patients attending the hospital. With a continuing backlog of patients and staff absences due to illness or self-isolation, there is a need to utilise technologies to reduce the number of face to face appointments. Patients undergoing amblyopia treatment account for a significant proportion of attendances in the orthoptic clinic so there are a number of methods being evaluated to determine if follow up appointments can be conducted remotely or the period of time between appointments can be extended.

There are numerous vision testing apps available for use on smart phones but the Royal College of Ophthalmology made a statement regarding the use of apps in paediatric ophthalmology during the COVID-19 pandemic in 2020, advising caution in their use due to the lack of evidence regarding safety and efficacy [1]. Since that publication, there have been studies evaluating the assessment of visual acuity (VA) by the parents/patients' using apps [2–4], printed or computer based tests [5, 6]. During a period of lockdown due to COVID-19 Painter et al. [7] evaluated the use of vision testing apps at home with instructions provided on how to test vision; however, they found that the engagement with this method was low, with less than 15% of participants providing a measurement. Online assessment through a live consultation with the clinician providing instructions and support throughout has been reported to have better results than parent assessment with written instructions [8, 9]; however, this still necessitates the involvement of a clinician. This means that outpatient appointments at the hospital may be reduced but not the level of clinical input.

The Amblyopia tracker app has been developed with the aim of being a tool for parents to monitor changes in vision at home during amblyopia treatment, rather than a replacement for clinician led VA testing. This app presents a single size optotype (0.0logMAR at 3 m) and instructs the parent to move closer or further away depending on the child’s response. It is designed to be used on a weekly basis by parents to monitor changes in vision, with the potential to improve compliance if parents can see changes in vision between appointments, reduce the frequency of appointments for those whose vision is improving and prioritise the patients whose vision is either not changing or even reducing.

Initial evaluation of the Amblyopia tracker app showed similar test retest variability to the iSight app and good agreement between the measurements [10]. This was under strict testing conditions in the hospital clinic performed by orthoptists which allows evaluation of the testing method of the app but evaluation is required by parents testing in the home environment to determine whether accuracy is maintained. Therefore, the aims of this phase of the study were to evaluate the feasibility and repeatability of parents testing their children at home and then to compare home test results to an assessment in clinic by an orthoptist.

## Methodology

Ethical approval was obtained from the HRA ethics committee and local approval through the Manchester Royal Eye Hospital Research and Development department. This study aligns with the conditions of the Declaration of Helsinki.

Patients who met the following inclusion criteria were identified from an online database:

- Under 18 years old
- Able to complete a crowded logMAR vision test without a matching card
- Interocular difference of ≥ 0.2 logMAR
- Visual acuity in the worse eye of 1.0 logMAR or better
- English speaking
- Refractive adaptation of 16 weeks completed if glasses worn
- Adult tester using iPhone with iOS 13 or above, or Android less than 5 years old

Those with accommodative inability due to accommodative dysfunction, aphakia, pseudophakia or atropine occlusion therapy were excluded on the basis that they would not be able to accommodate appropriately to a target varying between 15 and 300 cm.

Potential participants were identified through health record searches and were invited to participate via telephone call; informed consent was collected via email. Consenting parents/guardians received email instructions to download the Amblyopia tracker app. They were provided with a tape measure and instructed to complete the app tutorial before testing (as described previously [10]) which focuses on the setup only. There is an option for a trial run but this is not part of the automated setup to reduce the time requirements for the child. As all children were undertaking occlusion therapy, they were instructed to use their usual patches for testing. The app has both Kay pictures and letter optotypes; parents were instructed on which one to use based on what they did at their last hospital appointment. Optotypes were presented singly in a box with the distance from the optotype to crowding box being 2.5 times the stroke width [11, 12]. Optotypes were presented one at a time using an automated staircase with parents selecting if the response was correct or incorrect, and if three out of five optotypes were correct they moved to the next level. Testing started at 30 cm (equivalent to 1.0 logMAR) and if responses were correct, the test distance increased to a maximum of one metre (equivalent to 0.0 logMAR). Step sizes were on a logarithmic scale with each step resulting in a 0.1 logMAR change in acuity. One week following the delivery of the instructional email, parents were supplied with a link to an online (JISC) survey to gain an understanding of parents’ experience with the app and any problems encountered. The questionnaire consisted of four questions with a Likert scale, followed by a yes/no question on whether they would like to use the app in the future. In addition, all questions had an optional free text box to provide further details on their responses if they wished to do so.

Parents were instructed to perform one visual acuity test per day on any three days across a two-week period. Parents returned the vision scores by using the email mechanism within the app. Questionnaire results were stored on a password protected online database. A £20 retail voucher was emailed to the parent once all three vision scores and the questionnaire responses had been received.

After completion of the home testing phase, researchers confirmed with parents their participation in the final phase of the study via email or telephone call. For this phase, parents were instructed to perform one final home vision test 48 h either side of their next clinic appointment. At this appointment after the patient’s usual clinical orthoptic assessment was completed, one of the researchers completed a vision test using the Amblyopia tracker app and the iSight Pro app (method previously reported [10]). The iSight app was chosen to minimise the number of variables in the test method, as it uses the same optotypes (ETDRS letters and Kay pictures) presented on the same device [13]. The order of app test was randomised for each participant, with the weaker eye always tested first for each app. On completion, participants received a further £10 retail voucher.

### Statistical analysis

All distance data were converted to logMAR values for analysis. The iSight app scores were rounded to the nearest line (where a minimum of three out of five optotypes per line were correctly identified). Analysis is performed on responses from each eye and the interocular acuity difference (IAD). For the test–retest variability analysis, comparisons are made between the second and third tests to minimise any impact of lack of familiarity in the first test. The mean bias was calculated as the second test VA minus the third test VA, and for the comparison between tests, the Amblyopia tracker app VA was subtracted from the iSight VA. Limits of agreement were calculated as 1.96 times the standard deviation (SD) of the difference between the two tests. Comparisons of VA measures were performed using a paired *t*-test, with a value of < 0.05 considered as statistically significant.

## Results

### Home testing

There were 277 patients identified for recruitment. Ninety did not return phone calls or messages and a further 129 declined to participate either at the first telephone call or by not returning the consent form. Twenty-one participants withdrew during data collection. Thirty seven participants completed three measurements at home, mean age 6.8 years (SD 2.94), range 3.77–16.6 years, nine of which were under the age of five years and four participants were above the age of ten years.

Table 1 shows the VA values for the amblyopic and non-amblyopic eyes and the interocular acuity differences. Paired *t*-test showed for most tests a *p* value greater than 0.1, the only value below 0.1 was for the non-amblyopic eye comparing test one to test three (*p* = 0.055).

**Table 1:** Responses for each eye and the IAD on repeated testing. IAD is the magnitude of the difference between the two eyes, any negative values are changed to positive

	Amblyopic eye			Non amblyopic eye			Interocular acuity difference		
Test	1	2	3	1	2	3	1	2	3
Mean (SD)	0.39 (0.28)	0.41 (0.27)	0.46 (0.35)	0.1 (0.18)	0.13 (0.23)	0.14 (0.26)	0.29 (0.26)	0.28 (0.23)	0.32 (0.31)

Comparisons between all tests were made; the data in Table 2 are for the comparisons between the second and third test to minimise any impact of the participants being unfamiliar with the test. Data in the table show the difference of test two minus test three, meaning if numbers of participants who improved on the second test are equal to the numbers that got worse, the mean difference will be close to zero. Therefore the absolute difference has also been calculated, included in Table 2.

**Table 2:** Comparison of responses from the second and third tests

	Amblyopic eye	Non amblyopic eye	IAD
Mean of test 2 and 3 (SD)	0.41 (0.28)	0.13 (0.24)	0.29 (0.23)
Mean difference, test 2-test 3 (SD)	-0.006 (0.12)	-0.01 (0.07)	-0.05 (0.29)
Absolute mean difference, test 2- test 3 (SD)	0.08 (0.09)	0.04 (0.06)	
Lower limit of agreement	-0.24	-0.16	-0.6
Upper limit of agreement	0.23	0.13	0.5

**Fig. 1:**
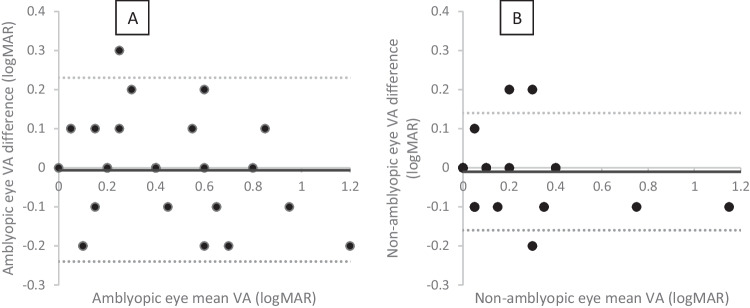
Bland Altman plot of the amblyopic eye (**A**) and non-amblyopic eye (**B**) comparing tests two and three. The solid black line indicates the mean bias and the dashed lines show the upper and lower limits of agreement

**Fig. 2:**
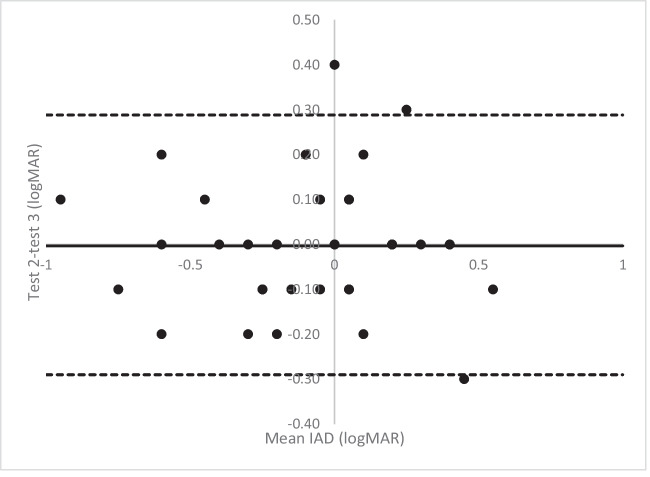
Bland Altman plot of the interocular acuity difference comparing tests two and three

In Figs. 1A, B and 2 and the mean bias is almost zero (*n* = 16 have a mean difference of zero on Fig. 1A and *n* = 25 on Fig. 1B, and *n* = 11 ± 0.1logMAR on Fig. 1A and *n* = 9 on Fig. 1B), indicating on average there is good agreement. On Fig. 1B, there is one child with a very poor VA result in the non-amblyopic eye (1.15logMAR) indicating potential testing difficulties. In Fig. 2, points above the line are for subjects where the score indicated a decrease in the IAD in the third test, points below the line indicated an increase in IAD on the third test. There are 71% of IAD measures less than or equal to 0.1 logMAR between the second and third test. Although the mean bias is close to zero there is some variation, including one data point indicating a difference in IAD of four logMAR lines. Looking at the profile for this outlier, it appears that the parent has mixed up the results for the right and left eyes resulting in this big difference.

### Parent evaluation

There were 34 completed questionnaires; responses of the likert scales are shown in Table 3. The three responders who indicated that testing was difficult or very difficult were all over the age of five years.

**Table 3:** Responses to the Likert scale questions, percentage of respondents with numbers of respondents in parentheses

Did you have any difficulty understanding the instructions?		How easy or difficult was it to carry out the test?		How willing was your child to do the test?	
Not at all	70.6% (24)	Very easy	61.8% (21)	Very willing	61.8% (21)
No, not really	26.5% (9)	Easy	26.5% (9)	Willing	29.4% (10)
Yes, a little	2.9% (1)	Neither easy nor difficult	2.9% (1)	Neither willing nor unwilling	2.9% (1)
Yes, some	0	Difficult	5.9% (2)	Unwilling	5.9% (2)
Yes, a lot	0	Very difficult	2.9% (1)	Very unwilling	0

The final question asked if the parents would use the app in the future if it became available to which 97.1% replied yes. In addition to the 34 responses included in Table 3, there were another ten responses from parents who did not complete the vision testing component. Evaluation of these responses did not indicate any reasons why the vision testing was incomplete, with many positive comments on the app and all ten parents saying they would use the app in the future.

Table 4 summarises the free text responses, some of which are reflective of all vision testing in children where engagement, concentration and willingness to wear a patch can be challenging. There were a number of comments related to the functionality of the app and how it could be improved. Other comments related to the impact on their attendance at the hospital with many benefits for the parent and child identified.

**Table 4:** Summary of common responses identified to the free text questions

Question	Responses	
	Positive	Negative
Did you have any difficulty understanding the instructions?	Easy to use Tutorial explained everything Appreciated the provision of a tape measure	Only one negative response “It wasn’t clear exactly where I measured from on the floor”
How easy or difficult was it to carry out the test?	Multiple comments about the ease of use Quick to complete so child was engaged with the test	The app was easy to use but testing their child was the bigger challenge Had to be on their knees to be on an eye level with their child There were some technical issues with the app
How willing was your child to do the test?	Some comments about their child being compliant/familiar with vision tests which may have made it easier Some parents turned it into a game to aid cooperation	Getting their child to sit still was a challenge, impacting on the test distance Having distractions, such as siblings, at home made it more difficult
Would you use the app in the future if it became available?	Benefits identified: • Less visits to the hospital • Can be done outside school hours • No child care required for siblings • Potential to monitor between hospital visits • Could build up concentration for hospital tests • Could be tested even if self-isolating • More manageable around careers and home commitments	Disadvantages: • Light reflecting on the screen • Noticeable to the child when they got one wrong • Felt too hard due to child’s reluctance, guessing and moving during testing

### Comparison of home and clinic testing

For this phase of the study, there were 23 participants, mean age 6.72 years (SD 2.60).

Table 5 shows the VA values for the Amblyopia tracker app measurements, at home and in the clinic, and the iSight app measurements. Comparisons of the home result to the clinic result and the iSight measurement showed no statistically significant differences (paired *t*-test, *p* > 0.3 in all cases). Comparing the home to clinic measure using the Amblyopia tracker app, the mean differences (SD) were amblyopic eye -0.004 (0.15) logMAR, non-amblyopic eye 0.00 (0.13) logMAR and IAD 0.004 (0.16). When comparing the home measurement to the iSight measurements, the mean differences (SD) were amblyopic eye -0.004 (0.16), non-amblyopic eye 0.013 (0.15) and IAD -0.07 (0.39). Comparing the Amblyopia tracker app measurement in clinic to the iSight app, the mean differences were amblyopic eye -0.00 (0.09) logMAR, non-amblyopic eye 0.013 (0.11) and IAD -0.08 (0.35) logMAR.

**Table 5:** VA values for the three measurements, home and clinic results being from the Amblyopia tracker app, with IAD presented as the magnitude of difference irrespective of which is the better-seeing eye

	Amblyopic eye			Nonamblyopic eye			Interocular acuity difference		
Test	Home	Clinic	iSight	Home	Clinic	iSight	Home	Clinic	iSight
Mean (SD)	0.3 (0.25)	0.3 (0.24)	0.3 (0.24)	0.07 (0.12)	0.07 (0.08)	0.05 (0.13)	0.26 (0.24)	0.24 (0.22)	0.25 (0.22)

**Fig. 3:**
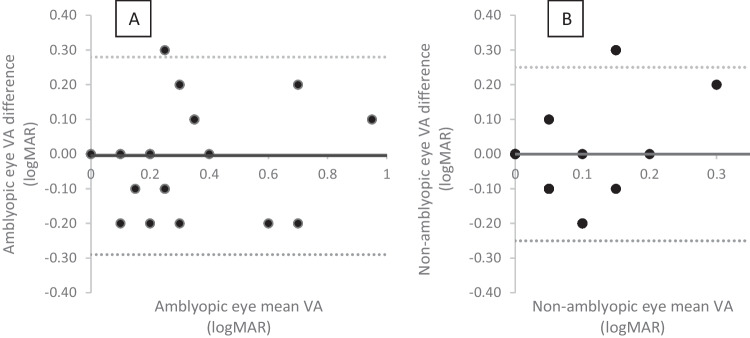
Bland Altman plot of the amblyopic eye (**A**) and non-amblyopic eye (**B**) comparing the home and clinic measurements using the Amblyopia tracker app

**Fig. 4:**
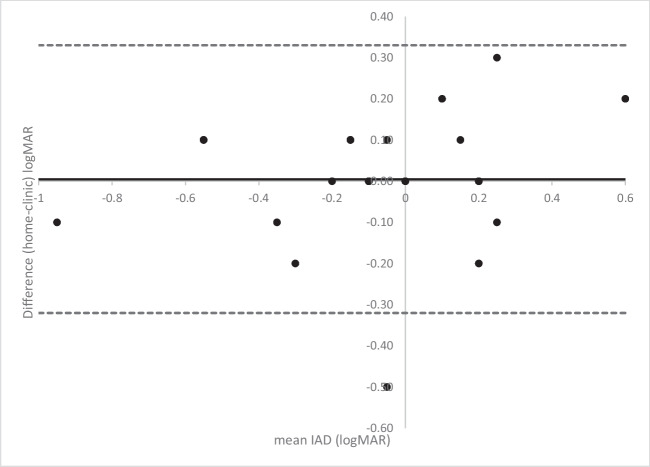
Bland Altman plot of the interocular acuity differences comparing the home and clinic measurements using the amblyopia tracker app

Figures 3A, B and 4 all show a mean bias close to zero but an increase in the limits of agreement, which are wider for the amblyopic eye than the non-amblyopic eye. Figure 4 shows six measures of the IAD being two logMAR lines or more different between tests. It also includes an outlier where the IAD is five lines difference between the tests. This child had acuities of 0.00 right eye and 0.3 left eye at home and 0.2 right eye and 0.0 left eye in clinic (with the same on the iSight test), suggesting the parent has mixed up the right and left results.

## Discussion

The findings from this study show that the Amblyopia tracker app when used by parents at home on repeated testing demonstrated no statistically significant differences. In addition, there were no significant differences between the home test by a parent and the test by a clinician using either the Amblyopia tracker app or the iSight app. Feedback from the parents was positive, with some helpful suggestions for ways to improve the app and how it is used.

While there is good agreement overall between the repeated home measures and the clinic measures using the Amblyopia tracker app (demonstrated by a mean bias close to zero), there are outliers of two lines or more, which would be considered a clinically significant difference. A difference of more than 0.1 logMAR occurred in 8/23 amblyopic eyes and 4/23 non-amblyopic eyes. Analysing these outliers, it appears that at least some are due to the parent mixing up the right and left eye results. This suggests the need for greater clarity and prompts in the app to prevent this from happening and supports the rationale for repeated testing. Previous studies have also suggested further tuition from the clinician either in person or via video conferencing may be beneficial in improving both the reliability and future participation from parents [7]. In addition, it is critical to ensure that the patients using home screening are engaged in the process and a shared approach to decision-making with regards to the management is undertaken [14]. However, the presence of outliers of two or more lines, which is considered to be a clinically significant change, could also suggest a degree of inaccuracy in the test procedure. The numbers of outliers on repeated testing with home testing is higher than with clinic testing, with the proportion of subjects with one or no lines difference between tests previously reported as 81% for the tracker app and 84% for the iSight app [10].

The biggest challenge for this study was recruitment with a response rate of 20% for home testing despite extensive efforts to recruit more participants, which included a financial incentive. Although it was challenging it is not unique to this study, with an average refusal rate for involvement in telehealth research reported at 32% (range of 4–89.9%) [15, 16] and Painter et al. [7] reported a recruitment rate of 14.6% for vision testing at home during the pandemic. Recruitment commenced over the winter period of 2020, and it was hypothesised due to the unpredictability of the Covid-19 Pandemic, parents may have been too busy with navigating school/childcare guidelines to take the time to participate in the study. It was anticipated that asking parents to undertake testing during a different time may have increased participation. However, it was attempted to contact parents again during the following year when restrictions eased, uptake from these parents did not improve. While we did not pursue reasons for refusal as part of our project aims, some volunteered a reason which included too busy due to childcare, issues with smartphone, and that they do not use apps.

The difficulties parents encountered and the reason for not participating further, whether at the consent stage or during data collection, suggests there is a requirement for additional support. Remote testing paradigms that have reported more successful engagement have been in the format of a virtual appointment, where the clinician instructs the parent and patient, and observes the child’s response [8, 9]. While the video conferencing approach may be beneficial, the aims of home testing of visual acuity need to be considered. During the Covid-19 pandemic, it may have been feasible to video conference call parents during clinic time to complete the home testing due to cancellation of clinics. This would have also avoided patients coming into the hospital unnecessarily later on in the pandemic. However, moving forward, home testing of visual acuity has the ability to potentially reduce costs, waiting times and potentially increase clinical outcomes due to the patient centred approach [17]. Therefore, support may come in the format of a clinician reviewing results from the home assessments on a regular basis to provide clinical input where required and to reassure parents that significant changes or concerns will be flagged up appropriately prior to the patient's next clinical visit. Given the outliers with significant variation between home testing and clinic, this level of support may provide reassurance for both the clinician and the parent/carer. Evaluation of a measurement undertaken at home involves multiple variables in comparison to a standard clinical setting. Testing by a family member within the clinical setting has been shown to have a modest correlation with 65.3% being within one line of the HOTV-ATS result [18]. Comparing the home and clinic measures using the Amblyopia tracker app, 61% had an IAD of one line or less, suggesting that testing at home also increases the variability in responses.

In conclusion, the Amblyopia tracker app has the potential to monitor changes in vision at home during amblyopia treatment, providing the parent is well educated and supported on the use of the app. For home testing to be successful, patient and clinician engagement is critical. Therefore, discussions are required to ensure the patient feels confident in their own ability and the app itself. This will require the parent to be fully educated on not only how to use the app, but the implications and the clinical importance of complying with the process.
